# Immunohistochemical analysis of RHAMM expression in normal and neoplastic human tissues: a cell cycle protein with distinctive expression in mitotic cells and testicular germ cells

**DOI:** 10.18632/oncotarget.24939

**Published:** 2018-04-20

**Authors:** Yao-Tseng Chen, Zhengming Chen, Yi-Chieh Nancy Du

**Affiliations:** ^1^ Department of Pathology and Laboratory Medicine, Weill Cornell Medicine, New York, 10065 NY, USA; ^2^ Division of Biostatistics and Epidemiology, Department of Healthcare Policy and Research, Weill Cornell Medicine, New York, 10065 NY, USA

**Keywords:** RHAMM, human tissues, normal, neoplastic, testicular, Pathology

## Abstract

Expression of Receptor for Hyaluronic Acid Mediated Motility (RHAMM) increases cellular motility and RHAMM overexpression promotes invasive phenotype and metastasis of cancer cells. RHAMM has been suggested as a biomarker for poor prognosis in several tumor types, including lung, breast, colorectal, gastric, pancreatic ductal, and ovarian cancers. RNA studies showed restricted RHAMM expression in normal tissues, but its protein expression data in tissues were limited. In light of its potential as a prognostic marker and a therapeutic target, we performed immunohistochemical analysis to systematically characterize *RHAMM* expression in normal and neoplastic human tissues. Among 29 normal adult tissues, RHAMM protein showed restricted expression and was observed in the thymus, lymph node/tonsil, small intestine, colon, skin, bone marrow, placenta, and testis. The cellular distribution patterns of RHAMM in these normal tissues were consistent with RHAMM being a G2/M cell cycle protein, and this was further supported in comparison to the expression of cyclin B2, another G2/M protein. However, unlike the subcellular localization of cyclin B2, RHAMM decorated mitotic spindles in both anaphase and metaphase. RHAMM expression in tumor tissues is variable; and higher RHAMM protein expression is associated with histologically higher-grade tumors in general. Distinct from its expression in somatic tissues, RHAMM showed diffuse, strong, stage-specific expression in the spermatocyte stage of germ cells in adult testis. The neoplastic counterpart, spermatocytic tumor, also showed strong RHAMM expression. This unique expression in testis suggests that RHAMM may function during normal testicular germ cell maturation.

## INTRODUCTION

Receptor for Hyaluronic Acid Mediated Motility (RHAMM, CD168) was initially identified as a protein that binds to hyaluronic acid (hyaluronan or HA) [1]. Hyaluronic acid is a simple glycosaminoglycan (a class of negatively charged polysaccharides) and is a major constituent of the extracellular matrix [2]. Increased production of hyaluronic acid has been correlated with increased migration and invasion in aggressive cancers.

RHAMM is believed to increase cellular motility through its interaction with the cytoskeleton [3]. Overexpression of RHAMM is sufficient to promote the invasive phenotype of cancer cells [4, 5]. Alternative mRNA splicing leads to four *RHAMM* isoforms, and we identified the gene product of *RHAMM isoform B (RHAMM^B^)* to promote liver metastasis of pancreatic neuroendocrine tumors [6]. We also demonstrated that 96% of metastatic non-small lung cancer expressed RHAMM proteins, and *RHAMM* mRNA expression correlated with shortened survival in lung adenocarcinoma [7]. Importantly, short hairpin RNA (shRNA)-mediated knockdown of *RHAMM* reduced the migratory ability of lung adenocarcinoma cells, suggesting that RHAMM is not only a prognostic factor but also contributes to lung cancer metastasis. Other studies have shown that RHAMM upregulation is a prognostic indicator for breast cancer, colorectal cancer, endometrial carcinomas, large cell lung cancer, gastric cancer, pancreatic ductal adenocarcinoma, and ovarian cancer [8–15].

RHAMM first appeared in vertebrates [16]. Previous studies have shown that high *RHAMM* mRNA was detected in testis, placenta, and thymus; very low *RHAMM* mRNA was detected in lung and pancreas, but not in other normal human tissues [17]. RHAMM protein expression in normal or tumor tissues, however, has not been well characterized. To further develop RHAMM as a prognostic biomarker and as a potential therapeutic target in cancer, we seek to define the cellular and subcellular distribution of RHAMM protein in normal and neoplastic human tissues.

## RESULTS

### RHAMM expression in normal tissues

**Figure 1:**
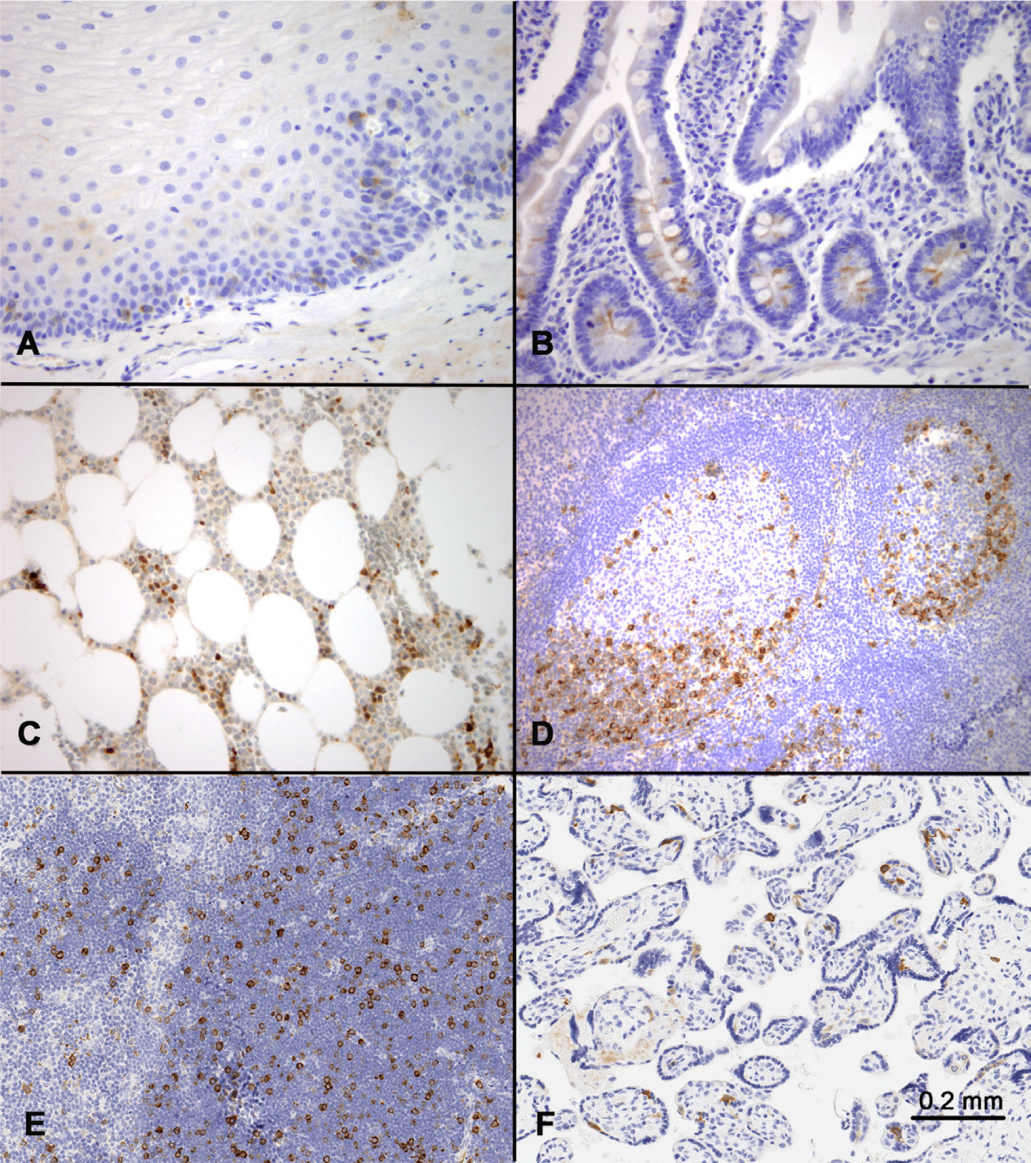
RHAMM expression in normal tissues Immunohistochemical staining identified scattered RHAMM-positive cells in (**A**) basal and parabasal cells of skin, (**B**) base of the crypts in small intestine, (**C**) bone marrow, (**D**) germinal centers in tonsil with a predominance in dark zones, (**E**) thymic cortex, and (**F**) placental trophoblasts. All positive cells showed cytoplasmic staining.

A panel of 29 normal human tissues was evaluated for RHAMM protein expression by immunohistochemistry. The specificity of the RHAMM antibody used was previously confirmed by showing reduced RHAMM protein levels in shRNA *RHAMM* knockdown cell lines in immunoblotting [7]. We found RHAMM protein expression to be restricted to the intestinal tract (small intestine and colon), skin, bone marrow, lymph node, tonsil, thymus, placenta, and testis (Figure 1 and Table 1). All 20 other tissues, i.e. heart, lung, kidney, cerebrum, cerebellum, pituitary, thyroid, adrenal, breast, salivary gland, esophagus, stomach, pancreas, liver, spleen, ovary, uterus, cervix, skeletal muscle, and prostate were negative for RHAMM expression.

**Table 1:** RNA and protein expression of RHAMM in normal human tissues

		RHAMM Expression	
Normal tissue type		RNA^*^	Protein
**Solid tissues**			
	adrenal	N.D.	0
	brain	0	0 (in cerebrum and cerebellum)
	breast	N.D.	0
	cervix	N.D.	0
	colon	0	scattered positive cells in the proliferative zones at base of crypts
	esophagus	N.D.	0
	liver	0	0
	lung	+	0
	heart	0	0
	kidney	0	0
	ovary	0	0
	pancreas	+	0
	pituitary	N.D.	0
	placenta	++	a subset of positive trophoblast cells in the full-term placenta
	prostate	0	0
	salivary gland	N.D.	0
	skeletal muscle	0	0
	skin	0	scattered positive cells in basal and parabasal layers
	small intestine	N.D.	scattered positive cells in the proliferative zones at base of crypts
	spleen	0	0
	stomach	N.D.	0
	thyroid	N.D.	0
	testis	++	most abundant RHAMM expression among all normal adult tissues, see text
	uterus	N.D.	0
**Hematopoietic and lymphoid tissues**			
	peripheral blood	0	N.D.
	bone marrow	N.D.	scattered positive cells
	lymph node/tonsil	N.D.	most of the positive cells clustered in the dark zones of the germinal centers; occasional positive cells in the paracortical areas
	thymus	++	positive cells abundant in the thymic cortex but absent in the medulla

^*^0: under detection limit; +: low level RNA expression; ++: high level RNA expression; RNA data summarized from [17]. N.D.: not determined.

In the intestinal tract and skin, scattered cells in the proliferative zones, i.e. the enterocytes at the bottom of the crypts and the basal and parabasal keratinocytes of the skin, showed weak to moderate positivity for RHAMM (Figure 1A and 1B). In comparison with epithelial tissues, hematopoietic and lymphoid tissues showed more prominent RHAMM protein expression (Figure 1C and 1D). In bone marrow, scattered positive cells were identified (Figure 1C). In lymph node and tonsil, although occasional RHAMM-positive cells were identified in the paracortical areas between germinal centers, most of the RHAMM-positive cells were clustered in the dark zones of the germinal centers (Figure 1D). In the thymus, RHAMM-positive cells were abundant in the thymic cortex but were absent in the medulla (Figure 1E). In the full-term placenta, RHAMM expression was detected in a subset of trophoblast cells (Figure 1F).

**Figure 2:**
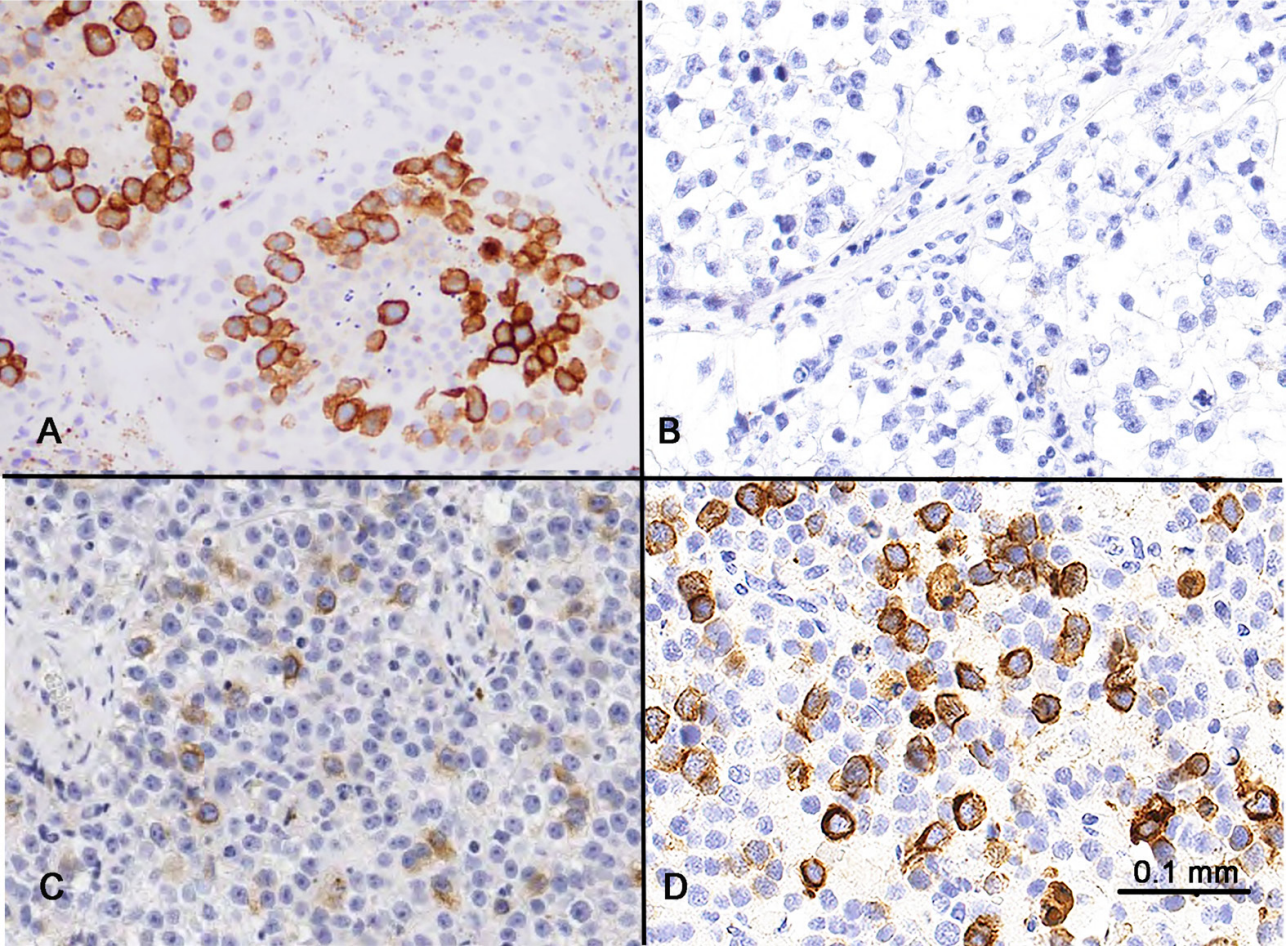
RHAMM expression in the testis and seminoma (**A**) Spermatocytes in the testis showed strong cytoplasmic expression. Weak expression was seen in the spermatogonia at the periphery of the seminiferous tubules. Spermatids and spermatozoa were RHAMM-negative. (**B** and **C**) Classic seminoma had variable RHAMM positivity, from negative (B) to up to 20% RHAMM-positive cells with weak to moderate intensity (C). (**D**) Spermatocytic tumors showed strong RHAMM staining in approximately 30% of the tumor cells.

Germ cells in adult testis showed the most abundant and consistent RHAMM expression among all normal adult tissues (Figure 2A). RHAMM expression was identified in germ cells and was maturation stage-specific, with all spermatocytes showing diffuse strong cytoplasmic staining, while only weak expression was observed in occasional spermatogonia, and none in the post-meiotic germ cells, i.e. spermatids and mature spermatozoa. Sertoli and Leydig cells showed no RHAMM expression.

### RHAMM expression in seminoma and spermatocytic tumor

The expression of RHAMM in germ cells prompted us to examine its expression in tumors with germ cell differentiation, including 66 cases of classic seminoma and 2 cases of spermatocytic tumor (known as “spermatocytic seminoma” in earlier literature and WHO classification). 61 of 66 cases (92%) of classic seminoma showed no (<1%, 33 cases) or low frequency (1–10%, 28 cases) of RHAMM-positive cells. 5 cases (8%) showed RHAMM positivity in 10–20% of tumor cells (Figure 2B and 2C). All positive cases exhibited cytoplasmic staining with low to moderate staining intensity. In comparison, both cases of spermatocytic tumor showed strong RHAMM staining in a higher proportion (~30%) of tumor cells (Figure 2D). One of the two cases had an intratubular (*in situ*) component, and a similar percentage, i.e. ~30%, of the tumor cells, was RHAMM-positive in this pre-invasive component.

Because the RHAMM antibody [EPR4055] recognized C-terminus of RHAMM, to confirm that no N-terminal variants of RHAMM existed in most of the seminomas, we used another RHAMM antibody [EPR4054], generated from a peptide immunogen of N-terminus. The staining using the EPR4054 antibody showed lower staining intensity in RHAMM-positive cells and led to a lower percentage of RHAMM-positive cells in tumors (Supplementary Figure 1). All subsequent experiments were performed with the EPR4055 antibody.

### RHAMM expression in non-germ-cell tumors

RHAMM expression in other tumor types was examined using commercially available tissue microarrays (TMAs) as well as whole sections in selected tumors. The tumors represented in the TMAs included carcinomas of breast, lung (small cell carcinoma, adenocarcinoma, squamous cell carcinoma), colon, kidney, pancreas, thyroid, liver (hepatocellular carcinoma, hepatoblastoma), cervix, uterus, skin (basal cell and squamous cell carcinomas) and nasopharynx, as well as pancreatic neuroendocrine tumors, lymphomas, meningiomas, and gastrointestinal stromal tumors.

**Figure 3:**
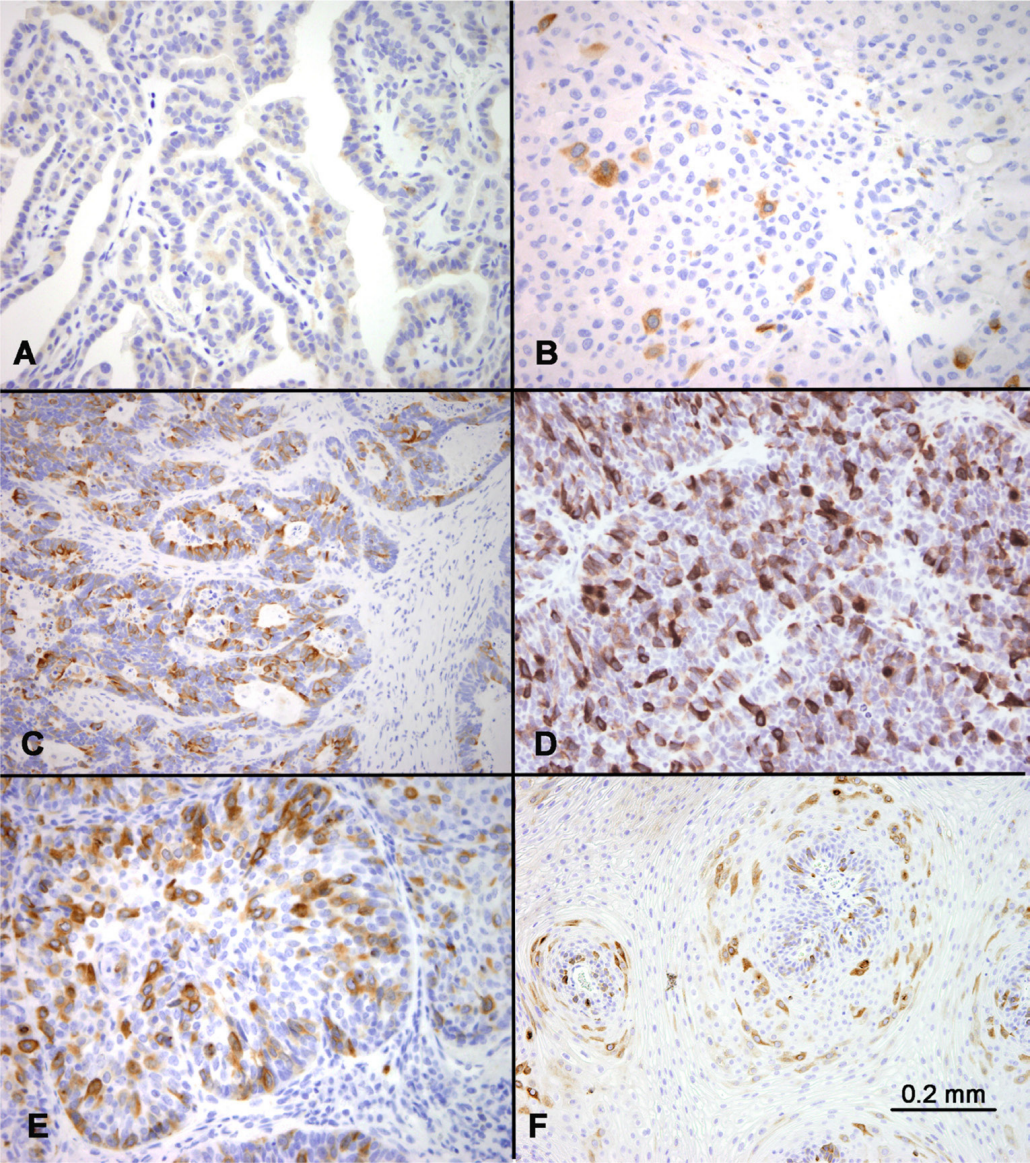
RHAMM expression in various tumors RHAMM expression was highly variable among different tumors. Examples of no or low expression included papillary thyroid carcinoma (**A**) and medullary thyroid carcinoma (**B**). In comparison, examples of abundant expression included a lung adenocarcinoma (**C**) and small cell carcinoma of the lung (**D**). Comparison of RHAMM in poorly-differentiated squamous cell carcinoma (of the lung) (**E**) and condyloma acuminatum (**F**) showed more abundant expression of RHAMM in the former.

No lineage specificity of RHAMM expression was observed. The percentages of RHAMM-positive cells varied widely among the tumor types, as well as among different tumors of the same histologic type. Benign neoplasms such as uterine leiomyoma and well-differentiated tumors such as papillary thyroid carcinomas showed no or very few RHAMM-positive cells (<1%) in the tissue cores analyzed (Figure 3A). In comparison, most malignant tumors showed RHAMM-positivity in 1–10% of tumor cells. These included low-grade neuroendocrine tumors such as medullary thyroid carcinoma (Figure 3B). A small subset of tumors, including lung adenocarcinoma (Figure 3C) and small cell lung carcinoma (Figure 3D), showed much higher percentages of RHAMM-positive cells, up to 50% of tumor cells in some cases. In general, the higher the histologic tumor grade, the higher the percentage of RHAMM-positive tumor cells, as illustrated by the comparison of poorly differentiated squamous cell carcinoma with condyloma acuminatum (Figure 3E and 3F). Of the 54 tumor tissues evaluated, histologic grades were available in 27 cases, and a statistically significant correlation between histologic grade and RHAMM expression was observed (*P* < 0.01, Table 2).

Table 2RHAMM protein expression correlates with higher tumor grade

**Table:** (A)

Case#	Organ	Tumor type	Grade^§^	RHAMM^*^
1	Uterus	Leiomyoma	0	0
2	Cerebrum	Atypical meningioma	0–1	1
3	Skin	Condyloma acuminatum	0–1	1
4	Pancreas	Adenocarcinoma	1	0
5	Thyroid	Medullary carcinoma	1	0
6	Kidney	Clear cell carcinoma	1	0
7	Uterus	Adenocarcinoma	1	0
8	Bladder	Leiomyosarcoma	1	0
9	Thyroid	Papillary carcinoma	1	1
10	Breast	Invasive ductal carcinoma	1	1
11	Colon	Adenocarcinoma	1	2
12	Cerebrum	Oligodendroglioma	2	0
13	Lung	Adenocarcinoma	2	1
14	Rectum	Adenocarcinoma	2	1
15	Cervix	Squamous cell carcinoma	2	1
16	Cervix	Squamous cell carcinoma	2	1
17	Bladder	Transitional cell carcinoma	2	1
18	Skin	Squamous cell carcinoma of chest wall	2	2
19	Ovary	Adenocarcinoma	2	3
21	Prostate	Adenocarcinoma	3	1
22	Esophagus	Adenocarcinoma	3	2
23	Small intestine	Adenocarcinoma	3	2
24	Ovary	Serous adenocarcinoma	3	3
25	Lung	Small cell carcinoma	3	3
26	Lung	Small cell carcinoma	3	3
27	Lung	Squamous cell carcinoma	3	3

^§^Tumor grade 0: benign; 0–1: Borderline neoplasm; 1–3, malignant from low to high grade.

^*^Scored 0 if <1% tumor cells positive; 1, 1–5% tumor cells; 2, 5–20% tumor cells; 3, >20% tumor cells.

**Table:** (B)

		RHAMM				
Tumor grade		0	1	2	3	
	Total					*P* value
0	1 (3.8)	1 (14.3)	0 (0.0)	0 (0.0)	0 (0.0)	0.001
0–1	2 (7.7)	0 (0.0)	2 (20.0)	0 (0.0)	0 (0.0)	
1	8 (30.8)	5 (71.4)	2 (20.0)	1 (25.0)	0 (0.0)	
2	8 (30.8)	1 (14.3)	5 (50.0)	1 (25.0)	1 (20.0)	
3	7 (26.9)	0 (0.0)	1 (10.0)	2 (50.0)	4 (80.0)	

Despite the differences in the frequency of RHAMM expression in different tumor types, RHAMM-positive cells were detected as isolated single cells scattered in a background of RHAMM-negative cells, and this spatial distribution pattern was highly consistent among tumors.

### RHAMM expression in mitotic cells

**Figure 4:**
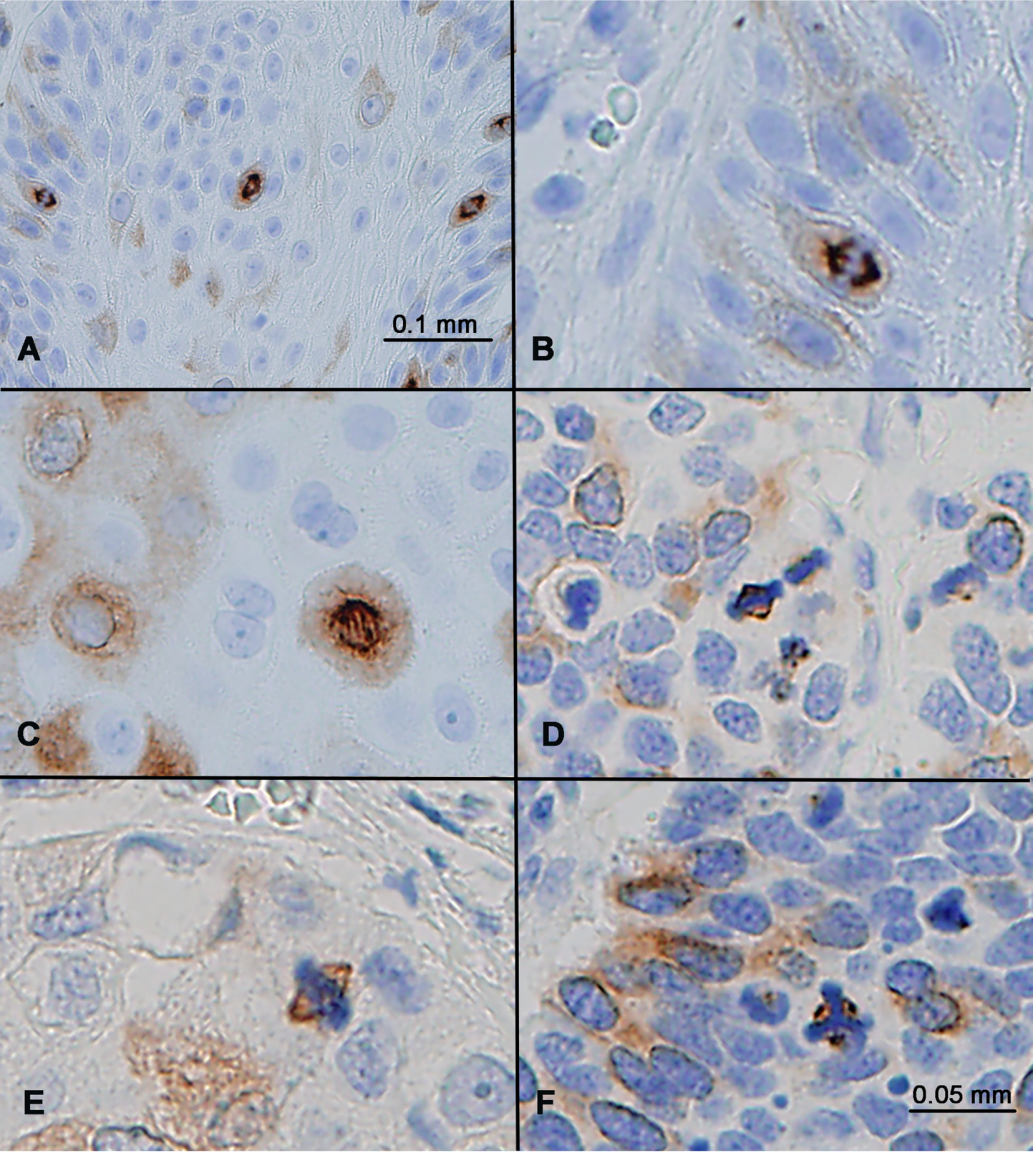
RHAMM expression in mitotic cells (**A**) Squamous cell carcinoma showed strong RHAMM staining of all mitotic figures, in addition to some cells with weak cytoplasmic staining. (**B**–**F**) RHAMM staining in mitotic anaphase (B, C), metaphase (D, E), and in a tumor cell with abnormal tripolar mitosis (F) in a small cell lung carcinoma.

RHAMM positivity manifested as diffuse cytoplasmic staining in non-mitotic cells, as illustrated in Figure 1 through Figure 3. No nuclear staining or membranous staining was evident. In addition to cytoplasmic expression, RHAMM expression was detected in all mitotic cells. In these cells, RHAMM was identified on mitotic spindles both in anaphase and in metaphase (Figure 4A–4E). In an abnormal tripolar mitosis in small cell lung cancer, RHAMM positivity was seen as three dots, suggesting its centrosomal localization (Figure 4F).

### Comparison of RHAMM and cyclin B2 expression

**Figure 5:**
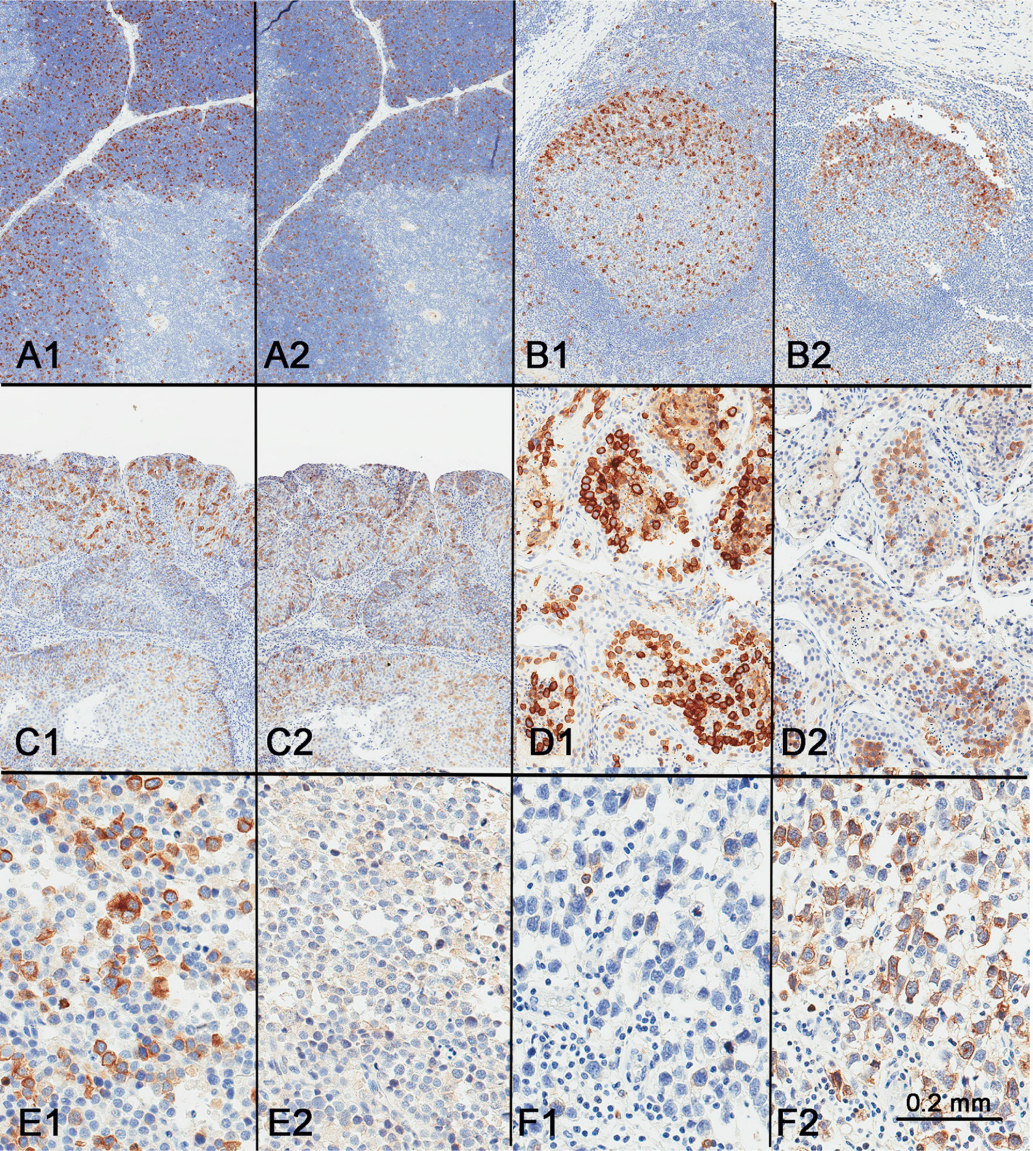
Comparison of RHAMM expression (A1–F1) with cyclin B2 expression (A2–F2) Similar expression patterns were seen in the thymus (A), tonsil (B), squamous cell carcinoma (C), and testis (D). However, spermatocytic tumor showed consistent and strong expression of RHAMM expression (**E1**) but not cyclin B2 (**E2**), while classic seminoma, generally weak for RHAMM (**F1**), showed strong expression of cyclin B2 (**F2**).

Since the above findings suggested that RHAMM is a cell cycle protein, we compared its expression pattern to that of cyclin B2, a cell cycle protein expressed in G2/M phases, using consecutive sections from the same tissue blocks. In normal thymus, tonsil, and squamous cell carcinoma, RHAMM-positive and cyclin B2-positive cells were similar in number and in distribution (Figure 5A–5C), with the exception of the absence cyclin B2 in mitotic cells.

As with RHAMM, cyclin B2 appeared to stain all or most spermatocytes in normal testis (Figure 5D). However, the staining patterns of RHAMM and cyclin B2 in seminoma and spermatocytic tumor were different. While RHAMM showed stronger expression in spermatocytic tumors than in any of the seminomas tested, cyclin B2 was only weakly expressed in spermatocytic tumors (Figure 5E). Seminomas showed variable expression of cyclin B2 (Figure 5F). There was no correlation in seminomas between the expression levels of RHAMM and cyclin B2 (Figure 5F).

**Figure 6:**
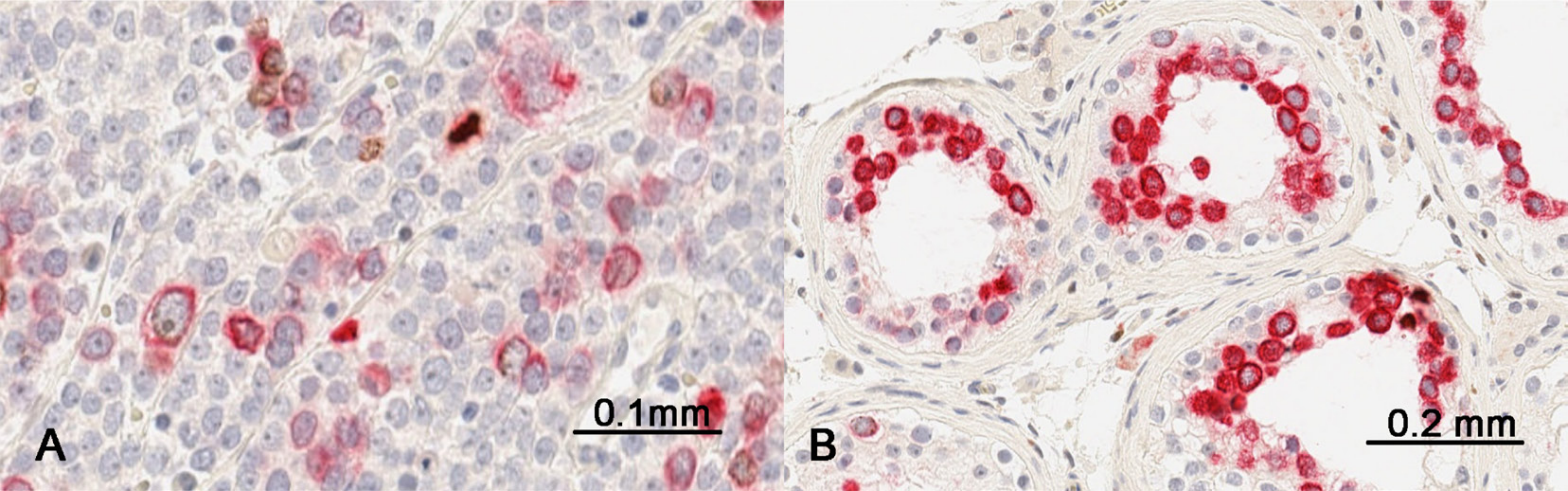
Comparison of RHAMM expression (red) with phospho-Histone H3 expression (brown) by double-immunostaining (**A**) a spermatocytic tumor and (**B**) non-neoplastic testis.

In addition, we performed double-immunostaining with antibodies against RHAMM and phospho-Histone H3 (Ser 10), a mitotic marker. Consistent with that RHAMM is a G2/M protein, mitotic cells constituted only a small proportion of RHAMM-positive cells both in spermatocytic seminoma and in normal testis (Figure 6). Seminoma tissue cores showed variable frequency of RHAMM-positive cells, but most of them were not in mitosis as shown in Figure 2C and 2D.

## DISCUSSION

Although the expression of *RHAMM* in normal tissues and some tumors has been examined at the mRNA level, there are only limited data in the literature on RHAMM protein expression in normal or tumor tissues in human [8–11, 13, 14]. Greiner *et al.* [17] used RT-PCR and found high *RHAMM* mRNA in testis, thymus, and placenta, very low *RHAMM* mRNA in lung and pancreas, but not in colon, skin, brain, heart kidney, liver, or prostate. In addition to demonstrating restricted RHAMM protein expression in testis, thymus, and placenta, we have shown RHAMM protein expression in intestine and skin, which had been considered RHAMM-negative in RT-PCR assays (Table 1). This discrepancy can be explained by the difference in assay sensitivity, since RHAMM-positive cells comprise less than 1%, if not <0.1%, of the cells in colon and skin, significantly less abundant than in thymus, testis, or placenta. We also demonstrated RHAMM protein expression in tonsil and bone marrow, which had not been previously examined. It is unclear why very low amount of *RHAMM* mRNA was detected in lung and pancreas by Greiner *et al.* [17].

Immunohistochemical analysis has allowed us to identify the location of RHAMM-positive cells in tissues and the subcellular localization of RHAMM. We found RHAMM protein expression to be restricted to organs with high cell turnover—thymus, lymph node, tonsil, small intestine, colon, skin, bone marrow, placenta, and testis. RHAMM-positive cells were present as isolated cells in the proliferative zones of these organs, such as in the basal layer of skin, the base of the intestinal crypts, and the germinal centers of the tonsils. No lineage specificity was observed in RHAMM expression, other than possibly in the testicular germ cells. These tissue and cellular distribution patterns suggest RHAMM as a cell cycle protein, which would be consistent with prior cell line studies that showed its mRNA and protein expression to be cell cycle-regulated [16, 18–20]. Interestingly, in the germinal centers of the tonsils, RHAMM protein was preferentially expressed in the dark zone than in the light zone. mRNA expression profiling of dark zone versus light zone has previously shown differential expression of multiple cell cycle proteins, including cyclin A2, B1, B2, and D3 [21], further supporting RHAMM as a cell cycle protein.

Previous studies showed *RHAMM* mRNA and protein expression to peak at G2/M [16, 18–20]. To explore this, we compared the distribution pattern of RHAMM with cyclin B2, a cell cycle protein known to function in the G2/M transition [20, 22]. Similar patterns of RHAMM and cyclin B2 staining were observed in thymus, testis, tonsil, and squamous cell carcinoma. Although we were not able to perform dual staining of RHAMM and cyclin B2 on the same tissue sections due to technical issues, our results from consecutive sections support RHAMM as a cell cycle protein in G2/M in human.

The pattern of RHAMM expression in testis is unique among non-neoplastic human tissues. All human spermatocytes show strong RHAMM expression, which is abruptly shut off in the post-spermatocyte germ cells, negative on all spermatids and spermatozoa. This pattern leads us to hypothesize that RHAMM may have a lineage-specific and stage-specific function in spermatogenesis beyond that of a cell cycle-regulated protein. To evaluate this possibility, we examined RHAMM expression in classical seminoma and spermatocytic tumor, and compared it with that of cyclin B2. Both tumor types originate in germ cells, but the former is malignant and is derived from prenatal primordial germ cells/gonocytes *in utero*, while the latter is almost always benign and originates in pre-meiotic adult germ cells, presumably at the spermatogonion or primary spermatocyte stage of maturation. Seminoma occurs in young adults and expresses markers typically seen in embryonic stem cells and primordial germ cells, including OCT4 and PLAP, while older patients develop spermatocytic tumors and have lost most markers of pluripotency, instead expressing markers characteristic of spermatogenesis, e.g. cancer-testis antigens [23]. The pattern of RHAMM expression we observed in classical seminomas was similar to that of other somatic cell tumors, with >90% of the cases showing no or low RHAMM expression and weak to moderate staining intensity. In contrast, spermatocytic tumors showed RHAMM expression in a higher percentage of tumor cells, with strong intensity similar to that seen in normal spermatocytes. Since spermatocytic tumor is a low-grade neoplasm, this consistent RHAMM staining, including the non-invasive intratubular component, supports the hypothesis that RHAMM might have a specific functional role during spermatogenesis. Cyclin B2, in comparison, showed lower expression in spermatocytic tumors than in some cases of seminoma, the opposite of RHAMM expression. Since spermatocytic tumors comprise of a variable degree of germ cell maturation, it is likely that a subset of tumor cells are equivalent in maturation to spermatocytes, resulting in *RHAMM* mRNA and protein expression. This proposed germ-cell specific function is highly relevant to the observation of atrophic seminiferous tubules and an increase in apoptosis in the testes in *RHAMM*-mutant [24] or *RHAMM*-deficient [25] mouse models, and explains the hypofertility in these mice. However, the proposal by Li *et al.* [24] that the carcinogenesis of seminoma is related to the “loss” of RHAMM expression is unfounded, as seminoma is derived from prenatal primordial germ cells, and not from the RHAMM-positive spermatocytes in the adult testis. Further studies are required to analyze RHAMM expression in prenatal primordial germ cells in order to assess its role in seminoma carcinogenesis.

Based on its expression in normal and tumor tissues, we propose that RHAMM has a specific role in spermatogenesis, but otherwise functions as a cell cycle-regulated cytoplasmic protein in adult human tissues. It is associated with the mitotic spindle as a cytoskeleton protein during mitosis in various human cell types. No membrane staining was identified in our immunohistochemical study, which suggests that the cell surface component of RHAMM is limited and below the detection threshold of our assay. The RHAMM staining patterns in this study are consistent with a recent suggestion that RHAMM functions as in the PLK1-dependent spindle positioning pathway [25]. Mice completely lost RHAMM expression have reduced survival with defects in fertility and brain structure. It is shown that mouse RHAMM acts at centrosomes to orient neural progenitor cell division.

We have previously demonstrated that overexpression of RHAMM isoform B significantly promotes metastasis of pancreatic neuroendocrine tumor in mouse models [6], and that there is a correlation between high histology grade and high RHAMM expression in non-small cell lung cancer [7]. We now confirm this correlation in other tumor types, and that RHAMM expression is low or absent in benign and low-grade tumors. In this regard, since RHAMM is a cell cycle protein that peaks at G2/M, it is analogous to other markers such as Ki-67 and may serve as a biomarker of active proliferation. Ki-67 proliferation index is currently used as a prognostic and predictive biomarker in invasive breast cancer, as well as a diagnostic marker in grading and classification of neuroendocrine tumors in the gastrointestinal tract. RHAMM has been suggested as a biomarker in colon cancer, since its expression was correlated with high histologic grade, tumor budding and poor survival [11]. Silencing of RHAMM was shown to reduce migration, proliferation, and metastasis of colorectal cancer cell lines [5]. RHAMM has also been proposed as a prognostic marker in gastric cancer based on the significant correlation between RHAMM expression and depth of invasion, nodal involvement, and vascular invasion [10]. However, in prostate cancer, Gust *et al.* showed that higher RHAMM expression was associated with negative lymph node status, lower T stage, and lower risk of (biochemical) treatment failure [26]. The reason for this apparent paradox in prostate cancer is unclear but may be attributable to the unproven specificity of the goat polyclonal RHAMM antibody used in that study, which has since been discontinued. The specificity of the RHAMM antibody used in our study has been proven by showing reduced RHAMM protein levels in shRNA *RHAMM* knockdown cell lines [7]. Additional studies using validated RHAMM antibodies are warranted to explore the potential clinical utility of RHAMM in prostate and other cancers.

## MATERIALS AND METHODS

### Tissues and tissue microarrays

Normal organ tissue microarrays (TMA), consisting of tissue cores derived from 24 human organs (cerebrum, cerebellum, adrenal gland, ovary, pancreas, lymph node, pituitary, testis, thyroid, breast, spleen, tonsil, thymus, bone marrow, lung, cardiac muscle, esophagus, stomach, small intestine, colon, liver, salivary gland, kidney, and prostate), was obtained from US Biomax (Rockville, MD, USA). Each tissue type was represented by tissue from 3 normal adult individuals, single core per specimen. In addition to the TMA, whole tissue sections from 5 additional normal tissues, including skin, placenta, uterus, cervix, and skeletal muscle were evaluated.

Cancer tissue microarrays with cancer-adjacent normal tissues, consisting of 54 specimens of malignant tumors from multiple organs, plus 18 cores of adjacent non-neoplastic tissues, was obtained from US Biomax (Rockville, MD, USA, Catalogue number: FDA808g-2). Each specimen was represented by a single core. In addition to the TMA, whole tissue sections from neoplastic tissues, including small cell lung carcinoma, squamous cell lung carcinoma, spermatocytic tumor, and condyloma acuminatum were evaluated.

A TMA was constructed in-house from a cohort of 66 cases of classic seminoma and one spermatocytic tumor, all resected at New York-Presbyterian Hospital/Weill Cornell Mediccal Center. This microarray was constructed using formalin-fixed, paraffin embedded tissue cores of 0.6 mm diameter, and each tumor was represented by three cores punched from morphologically representative tumor areas after histology review. Benign testis was incorporated into each microarray as controls.

The study was approved by the New York Presbyterian Hospital/Weill Cornell Medicine Institutional Review Board.

### Immunohistochemical analysis (IHC)

IHC was performed using a rabbit recombinant monoclonal RHAMM antibody [EPR4055] (Abcam, Cambridge, MA, USA) on paraffin embedded tissue sections on a Leica Bond system (Buffalo Grove, IL, USA) following the manufacturer's protocol. The sections were pre-treated using heat mediated antigen retrieval with Tris-EDTA buffer (pH = 9, epitope retrieval solution 2) for 20 min and incubated with RHAMM antibody (1:100 dilution) for 15 min at room temperature. RHAMM was detected using an HRP conjugated compact polymer system and DAB as the chromogen. Each section was counterstained with hematoxylin and mounted with Leica Micromount. RHAMM expression was scored as positive if any staining was present. The estimated percentage of RHAMM-positive cells was recorded and the staining intensity was characterized as weak, moderate or strong, and the cellular and subcellular location of the staining was noted.

For cyclin B2, IHC was performed using a rabbit recombinant monoclonal cyclin B2 antibody [R17985] (Abcam, Cambridge, MA, USA) on paraffin embedded tissue sections on a Leica Bond system (Buffalo Grove, IL.) following the manufacturer's protocol. Briefly, the section was pre-treated using heat mediated antigen retrieval with Sodium Citrate buffer (pH = 6, epitope retrieval solution 1) for 30 min and incubated with cyclin B2 antibody (1:100 dilution) for 60 min at room temperature. Cyclin B2 was detected using an HRP conjugated compact polymer system and DAB as the chromogen. Each section was counterstained with hematoxylin and mounted with Leica Micromount.

### Statistical analysis

The Cochran-Mantel-Haenszel (CMH) statistic was used to test a linear trend between tumor grades and levels of RHAMM staining. The test is two-sided with an alpha of 0.05 as the cutoff for statistical significance. All analyses were performed in SAS9.4 (SAS Institute, Cary, NC, USA).

## SUPPLEMENTARY MATERIALS FIGURE



## Abbreviations

RHAMM: Receptor for Hyaluronic Acid Mediated Motility
TMA: tissue microarray
IHC: Immunohistochemical analysis
