# Supplementary material for: Immunohistochemical analysis of RHAMM expression in normal and neoplastic human tissues: a cell cycle protein with distinctive expression in mitotic cells and testicular germ cells
Source: Oncotarget. 2018 Apr 20;9(30):20941–52. doi: 10.18632/oncotarget.24939 (PMC5940366; doi:10.18632/oncotarget.24939)
Supplement: Supplementary file 1 [file oncotarget-09-20941-s001.pdf]

Immunohistochemical analysis of RHAMM expression in normal and neoplastic human tissues: a cell cycle protein with distinctive expression in mitotic cells and testicular germ cells

SUPPLEMENTARY MATERIALS

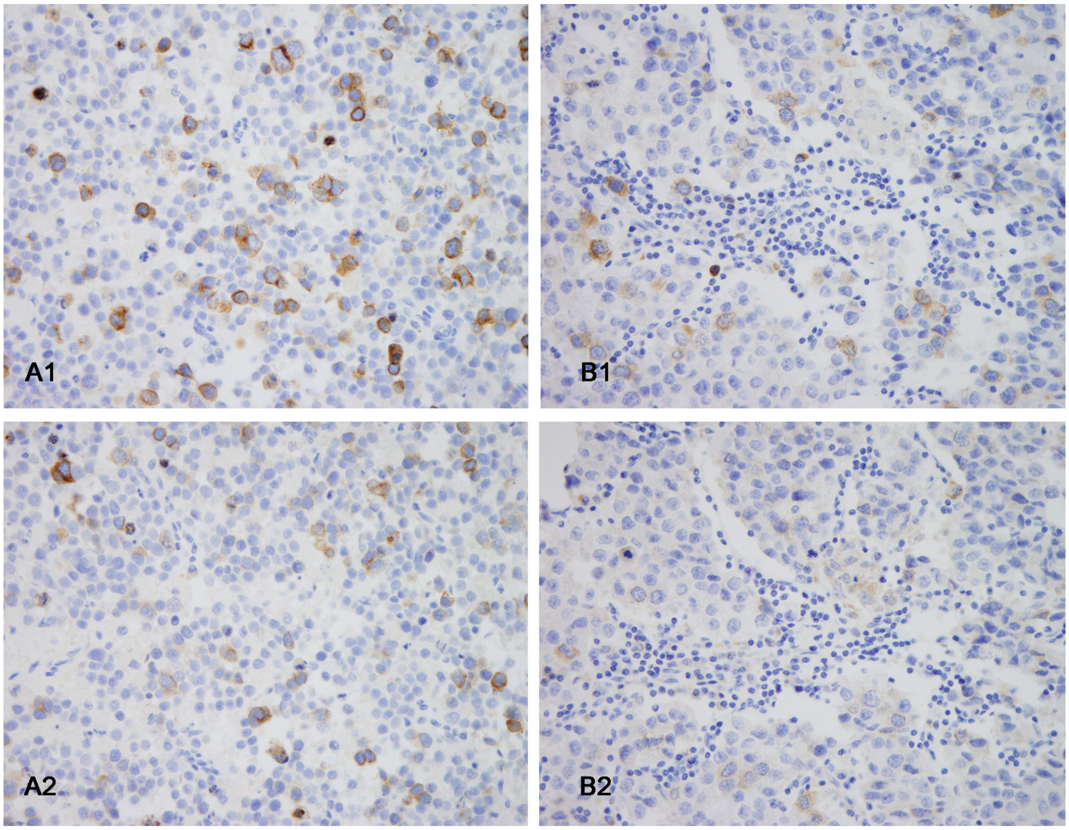

Supplementary Figure 1: The staining using the EPR4054 RHAMM antibody against N-terminus (Abcam, Cambridge, MA) did not show additional RHAMM-positive cells and had a lower percentage of RHAMM-positive cells in tumors than that using the EPR4055 RHAMM antibody. Upper panel, EPR4055 staining on a spermatocytic tumor (**A1**) and a seminoma (**B1**); Lower panel, EPR4054 staining on the same spermatocytic tumor (**A2**) and seminoma (**B2**).
